# Yeasts with Fermentative Potential Associated with Fruits of Camu-Camu (*Myrciaria dubia*, Kunth) from North of Brazilian Amazon

**DOI:** 10.1155/2021/9929059

**Published:** 2021-12-03

**Authors:** Ítalo Thiago Silveira Rocha Matos, Vanderly Andrade de Souza, Giovana do Rosário D'Angelo, Spartaco Astolfi Filho, Edson Júnior do Carmo, Marcos José Salgado Vital

**Affiliations:** ^1^Federal University of Amazonas, Av. Gal Rodrigo Otávio, 3000, Campus Universitário, Setor Sul, Manaus, AM, 69077-000, Brazil; ^2^Federal University of Roraima, Av. Ene Garcez, 2413, Aeroporto, Boa Vista, RR 69310-000, Brazil

## Abstract

Considering the high biotechnological potential of yeasts associated to edible fruits, a screening for these microorganisms, capable of alcoholic fermentation, was performed in ripe fruits of camu-camu (*Myrciaria dubia,* Kunth). The fruits were collected from north of Brazilian Amazon, in the floodplain of the Cauamé River. Yeasts were isolated, and fermentation capability was evaluated using Durham tubes. Quantitative assays were performed to calculate ethanol yield (g g^−1^), specific growth rate (h^−1^), and ethanol productivity (g L^−1^·h^−1^). Taxonomic identification was performed by ribosomal gene nucleotide sequence analysis by alignment using BLASTN. A total of fifteen yeast colonies were isolated, and three of them presented promising ability to ferment glucose to ethanol. These isolates were identified as *Candida orthopsilosis*, *Pichia kudriavzevii*, and *Meyerozyma caribbica*. When cultured in broth containing 180 g·L^−1^ of glucose, *M. caribbica* CC003 reached 91.7 percent of the maximum theoretical ethanol concentration (84.4 g·L^−1^), presenting an ethanol yield and productivity of 0.4688 g·g^−1^ and 0.781 g·L^−1^·h^−1^, respectively. These results indicate a promising potential of this isolate for bioprocess applications. This paper is a rare report of *C. orthopsilosis* with endophytic habit because most of the references indicate it as a human pathogen. Besides this, *M. caribbica* is a promising fermenter for alcoholic beverages due to its osmotolerance and high ethanol yield. This is the first paper reporting endophytic yeasts associated with fruits of *Myrciaria dubia*.

## 1. Introduction

Yeasts are microorganisms from the fungi kingdom that present asexual reproduction by fission or budding and with sexual reproductive structure not presenting fruiting bodies [1]. There are more than 2500 species described and recognized in the specialized literature [2], but despite this number, there are only about 80 species used in bioprocesses in the laboratory scale and a few more than twelve used in industrial processes [3].

Amazon is the greatest tropical rainforest of the world, containing about 220 edible fruit species [4], including camu-camu (*Myrciaria dubia*, Myrtaceae, Myrtales, Magnoliopsida, Magnoliophyta, Plantae). Fruits are potentially the natural habitat to a great variety of microorganisms because of their abundance of sugars and water, being favorable to proliferation of yeasts, mainly those capable of alcoholic fermentation. Despite all these potentials, research studies about yeast communities associated to Amazonian fruits are scarce, some of those dating more than two decades ago [5].

*Myrciaria dubia* has attracted a lot of attention because of its remarkable quantity of bioactive compounds [6]. Especially because of their antioxidant activity, their fruits have been used for functional food production, including an artisanal beer with high ascorbic acid concentration [7]. Besides this, there are no specific studies published about the microbiota associated with their leaves, roots, or fruits. The elucidation about yeasts associated to this plant may result in new strains with applicability to both biofuel and food industries.

In this context, for taxonomic purposes and for biotechnological applications, sampling efforts are a necessary approach. The aim of this work was to identify yeasts isolated from fruits of *Myrciaria dubia* capable of performing alcoholic fermentation and evaluate some kinetic parameters along the fermentative process.

## 2. Materials and Methods

### 2.1. Sampling and Yeast Isolation

Ripe fruits of *M. dubia* were collected manually from the bushes (Cauamé River floodplain, 2°51′54.79″N, 60°39′44.19″W) and maintained under refrigeration (4°C ± 2) until processing (about 2 h). The fruits were washed in fresh water to remove macroparticles, immersed in sodium hypochlorite solution (1% v/v) for 1 min and ethanol 70% for 1 min, and, finally, washed in sterilized distilled water for 2 min. After superficial decontamination, fruits were stored in a sterilized vessel at room temperature until developing an aroma resembling bread or alcoholic beverage at about 7 days (samples processed on the first day after they were collected did not present colony growth). Then, their peels were removed, and the mesocarps were mashed in sterilized distilled water (1 : 10 w/v).

Aliquots of 100 *μ*L were spread in plates containing GYMP agar (glucose 10 g·L^−1^, yeast extract 3 g·L^−1^, malt extract 3 g·L^−1^, peptone 5 g·L^−1^, agar 20 g·L^−1^, and pH 5.0), and the plates were incubated at 28°C until 48 h. Colonies presenting typical yeast's morphology were isolated and stored in sterilized mineral oil.

### 2.2. Fermentation Assays

A qualitative fermentation test was performed, evaluating the capability of the isolates to ferment glucose, D-xylose, sucrose, and maltose. A loopful of each isolate was inoculated in tubes containing liquid media composed of the respective sugar (40 g·L^−1^) and yeast extract (10 g·L^−1^). The tubes were incubated until 14 days at 28°C and evaluated every 24 h to identify fermentation, evidenced by gas retention into Durham tubes [8]. In these assays, the final cell concentration was equivalent to 1.0 in the McFarland scale, and they were performed in triplicate.

Some isolates were selected for a quantitative test, besides evaluating their osmotolerance, according to their ability to grow at high osmotic pressure (*π* = 24.68 atm), according to Jimoh et al. [9]. The isolates were inoculated in 250 mL Erlenmeyer flasks containing 100 mL of broth (pH 5.0) composed of glucose (180 g·L^−1^) and yeast extract (10 g·L^−1^) and incubated at 28°C and 120 rpm until 120 h. Fermentation was monitored every 12 h by measuring the mass of CO_2_ released, according to Dijck et al. [10]. Total inoculum was about 5 g·L^−1^ (dry weight), and kinetic parameters were evaluated according to Section 2.4.

### 2.3. Taxonomic Identification

Yeasts with fermentative capability were identified using the nucleotide sequences of D1/D2 26S rDNA genes. Genomic DNA was extracted according to the previous description [11] and amplified by PCR techniques using primers NL1 and NL4 [12]. The amplicons were used for the sequencing reaction using the BigDye kit (Applied Biosystems^®^), and nucleotide sequences were obtained using a 3500 automatic genetic analyzer (Applied Biosystems^®^). The sequences were compared to the GenBank database by alignment using the online tool BLASTN (https://blast.ncbi.nlm.nih.gov/Blast.cgi).

### 2.4. Kinetic Parameters and Analytical Methods

Ethanol concentration ([EtOH], *g* · L^−1^) along fermentation was determined by stoichiometric calculation, as previously described [10]. Specific growth rate (*μ*_MAX_, h^−1^) was calculated by the equation ln[EtOH]_*n*_/[EtOH]_*i*_=*μ*_MAX_*∗t* ± *b*, where [EtOH]_*n*_/[EtOH]_*i*_ is the relation between the current and initial ethanol concentration along the log phase and *t* is the time in hours, according to Duarte et al. [13].

Ethanol yield (Y_EtOH_, *g* · *g*^−1^) was calculated by the relation between the mass of ethanol produced and mass of glucose consumed. Ethanol productivity (Q_EtOH_, *g* · L^−1^ · h^−1^) was calculated by the relation between the final ethanol concentration and total time of fermentation before the stationary phase. Total reducing sugar concentration ([TRS], *g* · L^−1^) was determined using the dinitrosalicylic acid method (DNS). Aliquots of 20 *μ*L of the samples (diluted in distilled water, 1 : 10 v/v) were mixed with 180 *μ*L of distilled water and 300 *μ*L of DNS solution. After 5 min of boiling, 1500 *μ*L of distilled water was added, and the absorbance was determined at 540 nm. [TRS] was calculated using the formula obtained from a calibration curve.

## 3. Results

### 3.1. Isolation and Fermentative Capability

A total of 15 colonies were isolated, being identified with a numeric code preceded by “CC” (due to camu-camu, the vernacular form in Portuguese to *Myrciaria dubia*). More than a half were capable of fermenting glucose, and some were capable to ferment sucrose. No colonies presented capability to ferment D-xylose and maltose. The results are presented in Table 1.

Due to colony morphology and fermentative profile, some isolates were considered clones: CC001 and CC002; CC003, CC006, and CC007; and CC004, CC005, and CC015. Because of presenting positive results in all tubes of the qualitative assays, CC001, CC003, and CC015 were selected for quantitative fermentation assays.

### 3.2. Taxonomic Identification

A total of six isolates were identified, and the obtained sequences were deposited in the NCBI database under accession number PRJNA645409. The isolates were identified according to Table 2.

### 3.3. Kinetic Parameters and Analytical Methods

In assays with the initial glucose concentration of 180 g·L^−1^, *Pichia kudriavzevii* CC001 and *Candida orthopsilosis* CC015 presented the final ethanol concentration of 39.94 (±2.21) and 34.57 (±3.42) g·L^−1^, respectively. These results correspond, respectively, to 43.4 and 37.5 percent of the theoretical maximum; for this reason, they were considered not promising for fermentation applications and not further evaluated for other kinetic parameters.

**Figure 1:**
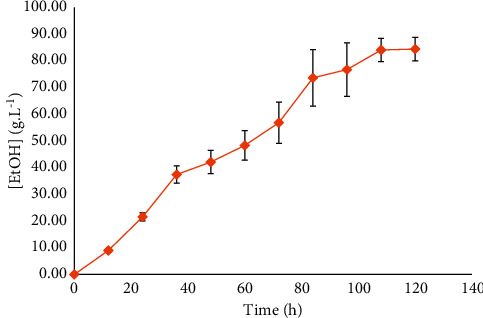
Ethanol production by *Meyerozyma caribbica* CC003 along 120 h of fermentation. The stationary phase was observed after 108 h. Error bars indicate standard deviation.

*Meyerozyma caribbica* CC003 presented a final ethanol concentration of 84.4 (±3.18) g·L^−1^, corresponding to 91.7% of the theoretical maximum. Final [TRS] indicated full consumption of glucose, meaning that this isolate is osmotolerant. Y_EtOH_ was 0.4688 g·g^−1^, Q_EtOH_ = 0.781 g·L^−1^.h^−1^, and *μ*_MAX_ = 0.025 h^−1^. The ethanol production kinetic by *M. caribbica* CC003 is presented in Figure 1.

## 4. Discussion

The occurrence of *P. kudriavzevii* was reported to be associated with blossom and ripped fruits of apple, pear, and plum in southwest Slovakia [14]. Its association with natural fermentation of ripped pulp fruits was described when microorganisms associated with *Ziziphus mauritiana* were screened [15]. Robs et al. [16] reported its occurrence associated with rotten fruits in a pineapple plantation in Rio de Janeiro, Brazil. Furthermore, the occurrence of this yeast was reported in association with rotten fruits of *Byrsonima* sp. [5], a common fruiting plant around the collecting site of this work.

*P. kudriavzevii* was also reported in association with natural fermentation of cereal dough in West Africa [17], in Chinese liquor Daqu [18], and as a component of the terroir of North Patagonian winemaking [19]. Together, these facts indicate that *P. kudriavzevii* is a typical endophytic yeast associated with fruits, including *M. dubia*, and safe for human consumption.

Most of the reports about *C. orthopsilosis* in scientific databases are related to human health problems. This species is closely related to *C. parapsilosis*, commonly described as a commensal in human skin and pathogenic yeast, capable to develop human tissue invasion and damage [20]. Scarce reports describe this yeast associated to fruits of *Opuntia stricta* [21] and tomatoes [22].

Its occurrence was described in natural fermentation of cotton seeds and rice beverage produced by Brazilian Amerindians [23]. This isolate could belong to the *C. parapsilosis* species complex, and further approach about its exact identification must be evaluated using other molecular markers. Because of the possibility of being a pathogenic isolate, its uses in industrial processes should be avoided until confirmative analysis for taxonomic identification and safe use.

Firstly, *Meyerozyma caribbica* was described as *Pichia caribbica*, distinguished from *P. guilliermondii* [24]. The type strain was isolated from sugar cane in Cuba, which explains its name “*caribbica.*” Further analysis repositioned it in a new genus, currently named *Meyerozyma* [25].

Its occurrence was reported in association with rhizosphere in high salinity soil in South Korea [26] and in corn-derived starch granules in Illinois, USA [1], and it was associated with different insects of the order Diptera [27]. *M. caribbica* was also reported as a prevalent species in the natural wet fermentation of coffee fruits and beans in Brazil [28] and in *Mangifera indica* fruits in Mexico [29]. These facts indicate that this species is worldwide distributed and presents a cosmopolitan habit.

The biotechnological applications of *M. caribbica* include its use as oleaginous yeast for biodiesel production [30] and as a biological control agent against *Colletotrichum gloeosporioides* in fruits of *Mangifera indica* [31]. The yeast powder presented effectiveness even after 6 months of storage. *M. caribbica* did not induce any animal toxicity or obvious cytotoxic activity [32]. It was used in a mixed inoculum with *Saccharomyces cerevisiae* to produce cachaça (sugar cane spirit) because of its ability to produce ethyl acetate and other compounds resembling the fruit's aroma [33]. Together, with its natural occurrence associated with fruits, it indicates that *M. caribbica* is safe to the food industry.

The results of qualitative fermentation tests corroborate as predicted by the literature, with most of the yeasts belonging to ascomycetous groups and capable of fermenting glucose [5]. The final ethanol concentration obtained by *P. kudriavzevii* CC001 was similar to that observed for different wild-type strains isolated by Chamnipa et al. [34]. The low yield observed can be explained by the relatively low incubation temperature as the highest yields were obtained when temperatures over 40°C were used [35, 36]. This possibility must be reevaluated in a further research project.

Ethanol production by *C. orthopsilosis* was similar to that obtained by Chamnipa et al. [34] when using a wild-type strain. Low yield in alcoholic fermentation seems to be a common feature for this species.

The percentage of the maximum theoretical yield observed for *M. caribbica* CC003 (91.7%) is higher than the yield (84.9%) obtained by Sukpipat et al. [37] when using a strain of *M. caribbica* for fermenting glucose (50 g·L^−1^) to ethanol. The values of Y_EtOH_ observed here were similar to those obtained when *S. cerevisiae* and *P. kudriavzevii* were used to produce ethanol using glucose as the substrate [38] and when *M. caribbica* was used to produce a new sweet sorghum distilled beverage [39]. Furthermore, Y_EtOH_ by CC003 is more than four times higher than that obtained when a wild-type strain of *M. caribbica*, isolated from Vietnam, was used to ferment glucose to ethanol [40].

Its ability to grow in moderate to high glucose concentrations and also present high ethanol yield indicates osmotolerance and suitability of this strain to ferment high original gravity brewing wort [41]. These results indicate that *M. caribbica* CC003 is a promising fermenter for application to both food and alcoholic beverage industries.

The values of Q_EtOH_ and *μ*_MAX_ were lower than all the compared references, indicating slow speed of fermentation probably due to relatively small initial inoculum. Temperature and initial inoculum are factors that need to be adjusted in further works.

## 5. Conclusions

This paper is a rare report of the endophytic occurrence of *C. orthopsilosis* as most of the references indicate this species as a human pathogen. There are scarce papers reporting the natural occurrence associated with fruits and other environmental samples. Other molecular markers must be evaluated to establish the difference between safe and pathogenic lineages of this species. Besides presenting high ethanol yield, *Meyerozyma caribbica* CC003 also presents tolerance to elevated amounts of glucose and ethanol. Therefore, it is a promising fermenter for alcoholic beverage production. The subsequent efforts will focus on the production of an alcoholic beverage using *M. caribbica* CC003, evaluating its chemistry profile, nutritional properties, and organoleptic characteristics.

## Figures and Tables

**Table 1:** Fermentative capability of yeasts isolated from *Myrciaria dubia*.

Isolate	Sugars			
	Glucose	Sucrose	Maltose	D-xylose

CC001	++	−	−	−
CC002	++	−	−	−
CC003	+++	−	−	−
CC004	+	+	−	−
CC005	+	+	−	−
CC006	+++	−	−	−
CC007	+++	−	−	−
CC008	−	−	−	−
CC009	−	−	−	−
CC010	−	−	−	−
CC011	−	−	−	−
CC012	−	−	−	−
CC013	−	−	−	−
CC014	−	−	−	−
CC015	+	+++	−	−

(+++): positive results in 48 h; (++): positive results in 72 h; (+): positive results in 96 h or more; (−): no fermentation evidence along 14 days.

**Table 2:** Taxonomic identification of yeasts isolated from *Myrciaria dubia*.

Isolate	Species	High similarity (%)	Strains related

CC001	*Pichia kudriavzevii*	100	ATCC 34135
CC002	*Pichia kudriavzevii*	100	ATCC 34135
CC005	*Candida orthopsilosis*	99.57	CBS 11337
CC015	*Candida orthopsilosis*	99.57	CBS 11337
CC003	*Meyerozyma caribbica*	99.81	CBS 9966
CC006	*Meyerozyma caribbica*	99.81	CBS 9966

## Data Availability

All the data generated or analysed during this study are included within this published article.
